# Validity of a new scoring system for assessment and decision guidance of misplaced pedicular screws

**DOI:** 10.1051/sicotj/2025015

**Published:** 2025-05-04

**Authors:** Mohamed El-Meshtawy, Moataz Abdelraheem Ahmed, Ibrahim El Sayed Abdellatif Abuomira, Amr Abdelhalem Amr, Mohamed A. A. Ibrahim

**Affiliations:** 1 Orthopaedic Department, Faculty of Medicine, Assiut University Yosri Raghib Street 71515 Assiut Egypt; 2 Orthopaedic Department, Faculty of Medicine, Al-Azhar University-Assiut 71524 Assiut Egypt; 3 Orthopaedic Department, Faculty of Medicine, Al-Azhar University-Cairo Al mokhayyam Al Dayem Street, Nasr City 11675 Cairo Egypt

**Keywords:** Pedicular screw, Pedicle cortical breach, MPSM score, Violation

## Abstract

*Background*: Pedicle screw fixation in the thoracolumbar spine has become more widely accepted with advancements in instrumentation and clinical efficacy have been made. The optimal way to interpret pedicle screw cortical breaches had the subject of a great deal of research. None of the previous classifications and grading systems include full neurological deficits that may result from screw misplacement and do not provide clear guidance for the management of screw violations, which is crucially needed in the literature. *Objectives*: Our study aimed to evaluate the reliability and validity of the use of a new scoring system (the Meshtawy Pedicular Screw Malposition – MPSM) for evaluating pedicle screw misplacement by a detailed clinical-radiographic comprehensive scoring system (MPSM) with sharp guidance for treating injurious violations by assessing the correlation between the neurological data of patients and computed tomography (CT) findings. *Patients and methods*: This prospective case series included 100 patients (508 pedicular screws) who underwent transpedicular fixation at Orthopedic department Al-Azhar University Hospital, Assiut branch, Egypt 255 (50.2%) screws were inserted on the right side, while 253 (49.8%) were inserted on the left side. Intra-observer reliability was examined by calculating Cronbach’s alpha intraclass correlation coefficient, which compares three measurements obtained by each observer at different time points. Inter-observer reliability was also examined by calculating Cronbach’s alpha intraclass correlation coefficient and comparing the average measurements obtained by each observer. *Results*: The MPSM demonstrated excellent (100%) intra-observer reliability for each observer regarding the violation score and total MPSM score. A strong positive and statistically significant correlation (Pearson test, *P* < 0.05) was found between severe neurological deficits and a greater degree of screw-pedicle violation. *Conclusion*: MPSM scoring is a valid and reliable system for evaluating pedicular screw violations and their possible neurological consequences in the thoracic and lumbosacral spine from D7 to S1. Moreover, grades obtained from the MPSM score are helpful for making clear decisions for management.

## Introduction

Although pedicle screws have been used extensively for fixation in thoracolumbar spine problems since the 1960s, improvements in instrumentation and clinical efficacy have occurred during the past 10 years [1].

The tiny size of the pedicles and the close proximity to neural components and important organs make the placement of thoracic pedicle screws more challenging and riskier than in the lumbar spine. A misplaced pedicular screw puts the lung, major vascular systems, spinal cord, and nerve roots at risk [2].

The optimal way to interpret pedicle screw cortical breaches has been the subject of a great deal of research; comparing study results becomes more challenging when these measurements are used in studies conducted by various universities because of their modest variations [3–5]. However, postoperative computed tomography (CT) scans, which are widely acknowledged as the most useful imaging technique for assessing pedicle screw correctness, are frequently needed [6–9].

The Gertzbein classification (1990) was initially used to evaluate screws positioned between T8 and S1. Since lateral screws were not included in his graded classification, the scale’s original purpose was to measure only the degree of spinal canal encroachment (grade 0 indicates no breach, grade 1 indicates less than 2 mm breach, grade 2 indicates between 2  and 4 mm breach and grade 3 indicates more than 4 mm breach) [3, 10].

This previous classification was slightly modified in a later study by Youkilis et al. to specify three different grades according to breach distance: grade 1 screws did not exhibit any signs of a pedicle breach, grade 2 screws breached 2 mm or less, and grade 3 screws breached more than 2 mm [4, 10].

According to the classification of Heary, each screw is assigned a grade: Grade I: the screw point is completely contained within the pedicle; Grade II: the screw breaches the lateral pedicle but is completely contained within the vertebral body (VB); Grade III is the tip penetrating the anterior or lateral VB; Grade IV breaches the medial or inferior pedicle; and Grade V breaches the pedicle or VB and puts the spinal cord, nerve root, or major arteries in danger and needs immediate revision. Overall, this grading system essentially emphasizes that screws that are close to vital structures (Grade V) should be removed immediately, but screws that break laterally but are still inside the rib may be acceptable (Grade II). This categorization system was also unique since it was the first to grade anterior breaches or those that passed through the vertebral body (Grade III). This scale’s limitation is that it does not account for the metric magnitude of violation in any direction; however, the Grade V classification, which is ultimately the most therapeutically important, slightly addresses this [5, 10].

None of the previously mentioned classifications and grading systems include complete neurological deficits that may result from screw misplacement, and they do not provide clear guidance for the management of screw violations, which is crucially needed in the literature. Our study objective is to present a detailed clinical-radiographic comprehensive scoring system (Meshtawy Pedicular Screw Malposition – MPSM) with sharp guidance for treating injurious violations.

## Patients and methods

This was a prospective study with a 3-month follow-up period that involved 100 patients who underwent transpedicular fixation at the Orthopedic department, Al-Azhar University Hospital, Assiut branch, Egypt between October 2021 and October 2022.

The inclusion criteria were neurologically free patients who underwent transpedicular screw fixation (TPSF) of the thoracic and lumbar spine.

The exclusion criteria were patients with spine deformity, e.g., scoliosis (congenital, idiopathic, or paralytic), that interferes with standard positioning of the TPSF and patients with preoperative neurological deficits.

### Administrative design

1. The purpose of the study and its advantages for the community were explained to each participant.
2. All participants provided written consent prior to being included in the study, and they were free to decline participation without it having an impact on their care.
3. All participant data were utilized exclusively for scientific research.
4. The privacy of the information gathered was guaranteed, and its use for purposes other than this research was prevented without consent.
5. The participants were informed of the researcher’s potential communication channels and asked if they had any more questions, and every participant was free to leave the study at any moment without having to give a reason.
6. The study’s findings were disclosed to every participant.
  Every patient had a complete history, a pre- and postoperative neurologic evaluation, and a CT scan.

### Ethical consideration

Our study followed the Helsinki declaration principles, and ethical approval was obtained from the committee of Al-Azhar University's Assiut branch college of medicine under registration number MD/AZ.AST./ORT017/19/184/5/2020.

### Methods of interpretation

The location of the screw within the pedicle was evaluated based on both neurological and radiographic assessments.

In every patient, postoperative CT was carried out to assess the implant location using 1 mm-thick CT slices and axial, coronal, and sagittal spine reformatting. The CT scan was performed within 48 h after surgery; unless deficits occurred, the CT was performed as early as possible, and every CT was performed in a soft copy (DICOM) folder and analyzed using a smart application for reading (DICOM) folder called RADIANT DICOM viewer version 2022.

Radiological assessment of screw position and violations was performed by three senior surgeons, and all patients were checked at three different times. The inter- and intra-observer reliability and correlation between the degree of pedicle violation and new neurological deficits were assessed using Cronbach’s alpha intraclass correlation coefficient if present.

#### Radiological assessment

**Figure 1:**
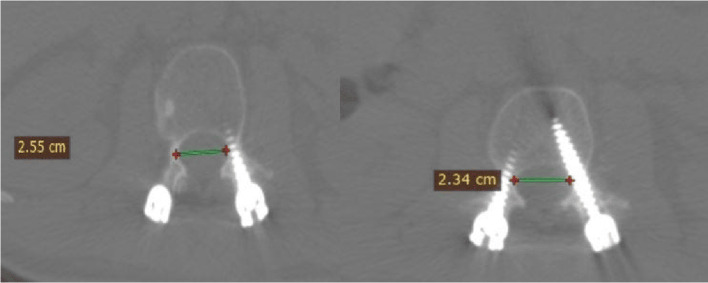
Canal encroachment.

**Figure 2:**
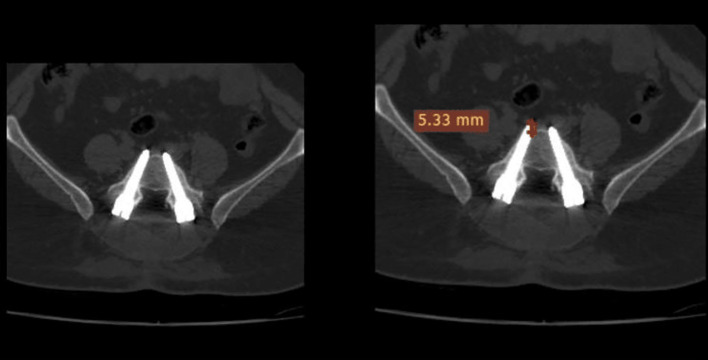
Violation length.

**Figure 3:**
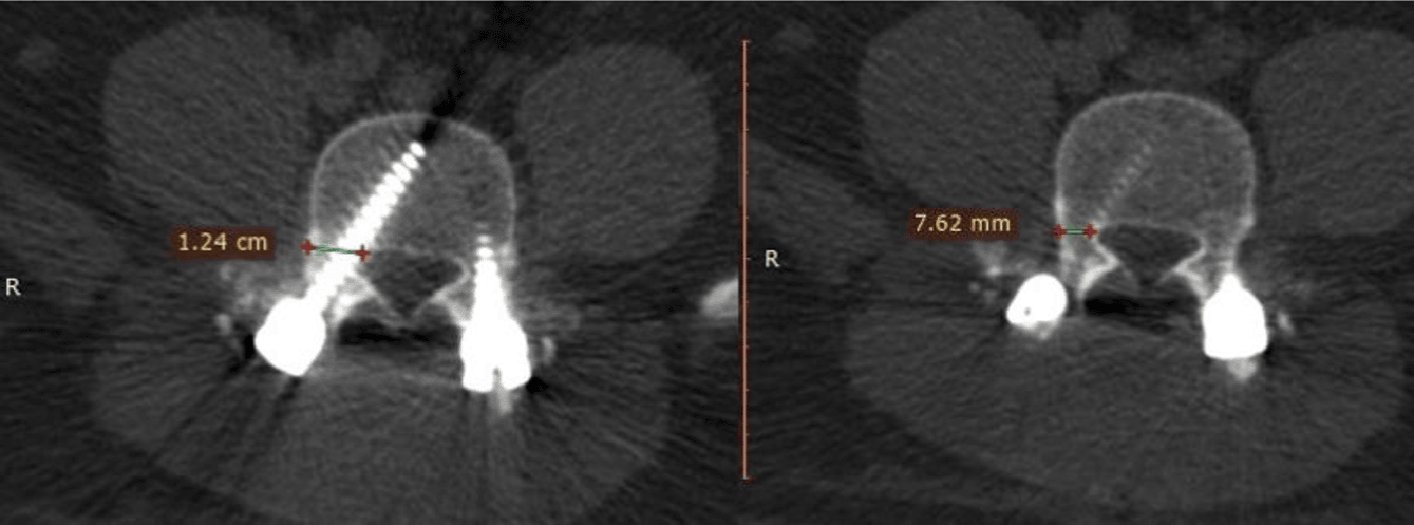
The width-pedicular width of the screw pedicle.

The following parameters were checked for each screw:

1. Error in screw insertion with respect to the pedicle angle (lateral, medial, superior, inferior, or anterior perforation).
2. The screw was placed inside the pedicle. The screw was regarded as (in) if the pedicle encircled it and if no part of the screw penetrated beyond the cortex. Or (out) if the screw was positioned incorrectly and some of it pierced the cortex. The scale on the CT image was used to measure screw displacement in millimeters (Figures 1–3).

The measurement of the length of the edge of the screw thread beyond the pedicle cortex allowed for further division of pedicle penetration into four categories (Table 1): zero (no violations), one point (≤2.0 mm), two points (2.1–4 mm), three points (>4.1–6 mm), and four points (>6 mm).

### Screw violation in any direction: Table 1

**Table 1:** Violation in mm and related points.

Points	0	1	2	3	4
Violation	No violation	≤ 2 mm	< 4 mm (2.1:4 mm)	<6 mm (4.1:6 mm)	>6 mm

#### Methods for the measurement of screw violation

- Canal encroachment: to assess medial pedicular wall violation (Figure 1).
- Length: used to assess anterior violation measured from the tip of the screw and anterior vertebral border. (Figure 2).
- The width of the pedicle and screw minus the pedicular width were used for assessing the medial, lateral, superior and inferior breaches, as shown in (Figure 3).

#### Neurological assessment: Table 2

Therefore, for every screw violation, 2 points were summed, one for the metric extent of cortical violation and the other for neurological deficits after the operation (Table 3).

**Table 2:** Neurological complication-related points.

	Neurological complication	Points
A	No deficit at all	0
B	Root affection	
	Sensory root deficit (pain ± hypoesthesia)	1
	Motor root deficit	2
	Mixed (sensory, motor)	3
C	Cord affection	
	Mono-paresis	4
	Paraparesis without sphincter affection	5
	Paraparesis with sphincter affection	6
	Complete paraplegia	7
D	Cauda equina syndrome	
	Incomplete without sphincter affection	4
	Incomplete with sphincter affection	5
	Complete cauda equina syndrome	6

**Table 3:** Grades of MPSM score.

Points	Grade	State of screw	Decision
≤2 points	Grade I	No or minor screw violation	Does not require revision
3–5 points	Grade II	Dangerous screw violation	May be revised
≥6 points	Grade III	Extremely dangerous screw violation	Must be revised

The MPSM score was used to correlate the CT results with the postoperative clinical data.

### Statistical analysis

SPSS version 26 (Chicago, IL, USA) was used for statistical analysis. Categorical variables are presented as percentages, and continuous variables, which are quantitative, are represented as the mean ± SD. Additionally, paired *t*-tests were used to compare continuous variables, and chi-square tests (χ^2^ tests) were used to compare categorical variables.

## Results

A total of 100 patients who underwent spine surgery were enrolled in our study. Table 4 summarizes the basic demographic data of the enrolled patients, including age, sex, spine pathology, level of pathology, and number of pathological levels.

**Table 4:** Baseline demographic data (*N* = 100 patients), (508 screw).

	Frequency	Percentage
Age (years)	Mean 42.4 ± 9.3 (Range, 22–64)	
Less than 30	12	12
30–40	32	32
41–50	41	41
More than 50 years	15	15
Gender		
Female	47	47
Male	53	53
Spine pathology		
Lumbar disc prolapse	51	51
Spondylolysis	30	30
Fracture	8	8
Posttraumatic kyphosis	2	2
Spinal tumour	2	2
Spinal infection	2	2
Failed spinal fusion	5	5
Number of pathological levels		
Single level	52	52
Two level	28	28
Multi-level	20	20
Level of spine pathology		
Dorsal spine	5	5
Lumbosacral spine	95	95

### Age and sex distribution

The average age of the included patients was 42.4 ± 9.3 years, ranging from 22 to 64 years. Patients were classified into four age groups: less than 30 years (*n* = 12), between 30 and 40 years (*n* = 32), between 41 and 50 years (*n* = 41), and more than 50 years (*n* = 15). In our study, 47 (47%) patients were males, whereas 53 (53%) were females.

### Spine pathology

As regards the type of spine pathology (Table 4), seven different spine pathologies were identified and treated, including lumbar disc prolapse (LDP) (*n* = 51), spondylolysis spondylolisthesis (*n* = 30), fracture (*n* = 8), post-traumatic kyphosis (*n* = 2), spinal tumour (*n* = 2), spinal infection (*n* = 2), and failed spinal fusion (*n* = 5).

### Level and number of spine pathologies

The number of pathological results is shown in Table 4. A single-level effect was reported in 52 (52%) patients, a two-level effect was reported in 28 (28%) patients, a multi-level effect was reported in 20 (20%) patients, the dorsal spine was involved in five (5%) patients, and the lumbosacral spine was involved in 95 (95%) patients, as shown in Table 4.

### Instrumentation

The type of surgical intervention used varied among participants and included decompression/discectomy/PLF (*n* = 37), PLIF (*n* = 46), TLIF (*n* = 5), TPSF (*n* = 8), Meshtawy transpedicular opening wedge osteotomy (*n* = 2), and corpectomy (*n* = 2). The characteristics of the surgical procedures are summarized in Table 5.

**Table 5:** Surgical procedure (*N* = 100 patients).

Type of procedure	Frequency	Percentage
Decompression/Discectomy/PLF	37	37
PLIF	46	46
TLIF	5	5
TPSF	8	8
MESH TWO	2	2
Corpectomy	2	2

PLF: posterolateral fusion; PLIF: posterior lumbar interbody fusion; TLIF: transforaminal lumbar interbody fusion; TPSF: transpedicular screw fixation; MESH TWO: Meshtawy transpedicular wedge opening osteotomy.

### Screws distribution

Regarding screw distribution, A total number of 508 screws were fixed in our study; 255 (50.2%) screws were inserted on the right side, while 253 (49.8%) were placed on the left side. Regarding the level of screw fixation, 12 different levels were identified, including D7 (*n* = 2), D8 (*n* = 1), D9 (*n* = 3), D10 (*n* = 6), D11 (*n* = 14), D12 (*n* = 16), L1 (*n* = 12), L2 (*n* = 32), L3 (*n* = 54), L4 (*n* = 117), L5 (*n* = 161), and S1 (*n* = 90). Characteristics of screw distribution are summarized in Table 6.

**Table 6:** Screw distribution (*N* = 100 patients, 508 screws).

	Frequency	Percentage
Side of screw fixation		
Right	255	50.2
Left	253	49.8
Level of screw fixation		
D7	2	0.4
D8	1	0.2
D9	3	0.6
D10	6	1.2
D11	14	2.8
D12	16	3.1
L1	12	2.4
L2	32	6.3
L3	54	10.6
L4	117	23
L5	161	31.7
S1	90	17.7

D: dorsal spine; L: lumber spine; S: sacral spine.

### Screw malposition

As regards the pedicle violation, it was associated with 117 (23%) screws, whereas 391 (77%) had no violation reported; it was reported associated with 48 (9.4%) screws for the medial cortex, 18 (3.5%) screws for the lateral cortex, 1 (0.2%) screw for superior cortex, 4 (0.8%) screws for inferior cortex, and 46 (9.1%) screws for anterior cortex.

The extent of screw violation, as measured by each observer, is shown in Table 7. The mean extent of medial violation was 3.4 ± 2.3, 3.8 ± 2.3, and 3.8 ± 2.3 mm, as measured by observers I, II, and III, respectively. The mean extent of lateral violation was 2.8 ± 1.5, 3.4 ± 1.8, and 3.4 ± 2.1 mm, as measured by observers I, II, and III, respectively. The mean extent of superior violation was 2, 2.1, and 2.2 mm, as measured by observers I, II, and III, respectively. The mean extent of IVC was 1.8 ± 0.9, 2.2 ± 0.8, and 2.1 ± 0.9 mm, as measured by observers I, II, and III, respectively. The mean extent of anterior violation was 4 ± 1.7, 3 ± 1.8, and 4.1 ± 1.7 mm, as measured by observers I, II, and III, respectively.

**Table 7:** Pedicle violation (*N* = 100 patients, 508 screws).

	Observer I	Observer II	Observer III	*P*-value
No violation	391 (77)	391 (77)	391 (77)	1.000*
Medial violation	48 (9.4)	48 (9.4)	48 (9.4)	0.475**
Mean ± SD	3.4 ± 2.3	3.8 ± 2.3	3.8 ± 2.3	
Range	0.4–12.7	1–12	0.9–12.5	
Lateral violation	18 (3.5)	18 (3.5)	18 (3.5)	1.000*
Mean ± SD	2.8 ± 1.5	3.4 ± 1.8	3.4 ± 2.1	0.662**
Range	0.4–5.6	1–9	1–9.8	
Superior violation	1 (0.2)	1 (0.2)	1 (0.2)	-
Mean ± SD	2	2.1	2.2	
Range	–	–	–	
Inferior violation	4 (0.8)	4 (0.8)	4 (0.8)	1.000*
Mean ± SD	1.8 ± 0.9	2.2 ± 0.8	2.1 ± 0.9	0.825**
Range	0.5–2.5	1–2.7	0.8–2.9	
Anterior violation	46 (9.1)	46 (9.1)	46 (9.1)	1.000*
Mean ± SD	4 ± 1.7	3 ± 1.8	4.1 ± 1.7	0.949**
Range	1–10	1–9	1–10	

*Chi-square test; **One-way ANOVA. This table shows that no statistically significant difference was found between the observers in terms of the direction or extent of screw violation.

### Grading of MPSM score

As shown in Table 8, safe screw violation (Grade I/no revision required) was reported in 439 (86.4%), 440 (86.6%), and 439 (86.4%) patients according to Observer I, II, and III, respectively. Dangerous screw violation (Grade II, revision may be required) was reported in 62 (12.2%), 61 (12%), and 62 (12.2%) patients according to Observer I, II, and III, respectively. Extremely dangerous screw violation (Grade III, must be revised) was reported in 7 (1.4%) patients according to all 3 observers. No statistically significant difference was found between observers in terms of MPSM grade (χ^2^ test, *P* = .931).

**Table 8:** Grade of MPSM score (*N* = 100 patients, 508 screws).

	Observer I		Observer II		Observer III		*P-*value
	No.	%	No.	%	No.	%	
MPSM total score							>.05
0 Point	404	79.5	403	79.3	403	79.3	
1 Point	18	3.5	20	3.9	20	3.9	
2 Point	17	3.3	17	3.3	16	3.1	
3 Point	30	5.9	29	5.7	30	5.9	
4 Point	27	5.3	28	5.5	27	5.3	
5 Point	5	1.0	4	0.8	5	1.0	
6 Point	3	0.6	3	0.6	3	0.6	
7 Point	3	0.6	3	0.6	3	0.6	
8 Point	1	0.2	1	0.2	1	0.2	
Grading							>.05
Grade I	439	86.4	440	86.6	439	86.4	
Grade II	62	12.2	61	12	62	12.2	
Grade III	7	1.4	7	1.4	7	1.4	

*Chi-square test.

### Reliability of the MPSM

Regarding intra-observer reliability, as shown in Tables 9–11, the MPSM demonstrated excellent (100%) intra-observer reliability with a confidence interval (95%) for each observer regarding the violation score and total MPSM score.

**Table 9:** MPSM score according to Observer I (*N* = 100 patients, 508 screws).

	1st measurement	2nd measurement	3rd measurement	Cronbach’s alpha correlation coefficient	
				Average	95% CI
Violation				1.0	1.0–1.0
Mean ± SD	0.48 ± 1.04	0.48 ± 1.04	0.48 ± 1.04		
Range	0–4	0–4	0–4		
Neurological examination					
Mean ± SD	0.16 ± 0.5				
Range	0–4				
Total score					
Mean ± SD	0.63 ± 1.4	0.63 ± 1.4	0.63 ± 1.4	1.0	1.0–1.0
Range	0–8	0–8	0–8		

**Table 10:** MPSM score according to Observer II (*N* = 100 patients, 508 screws).

	1st measurement	2nd measurement	3rd measurement	Cronbach’s alpha correlation coefficient	
				Average	95% CI
Violation				1.0	1.0–1.0
Mean ± SD	0.48 ± 1.04	0.47 ± 1.03	0.48 ± 1.04		
Range	0–4	0–4	0–4		
Neurological examination					
Mean ± SD	0.15 ± 0.5				
Range	0–4				
Total score					
Mean ± SD	0.63 ± 1.4	0.63 ± 1.4	0.63 ± 1.4	1.0	1.0–1.0
Range	0–8	0–8	0–8		

**Table 11:** MPSM score according to Observer II (*N* = 100 patients, 508 screws).

	1st measurement	2nd measurement	3rd measurement	Cronbach’s alpha correlation coefficient	
				Average	95% CI
Violation				1.0	1.0–1.0
Mean ± SD	0.48 ± 1.04	0.48 ± 1.04	0.48 ± 1.04		
Range	0–4	0–4	0–4		
Neurological examination					
Mean ± SD	0.15 ± 0.5				
Range	0–4				
Total score					
Mean ± SD	0.63 ± 1.4	0.63 ± 1.4	0.63 ± 1.4	1.0	1.0–1.0
Range	0–8	0–8	0–8		

### Inter-observer reliability

Regarding Inter-observer reliability, as shown in Table 12, the MPSM demonstrated excellent (99%) inter-observer reliability regarding the violation score and total MPSM score, with a confidence interval of 95%.

**Table 12:** MPSM score inter-observer reliability (*N* = 100 patients, 508 screws).

	Observer I	Observer II	Observer III	Cronbach’s alpha correlation coefficient	
				Average	95% CI
Violation				0.99	0.99–0.99
Mean ± SD	0.48 ± 1.04	0.48 ± 1.04	0.48 ± 1.04		
Range	0–4	0–4	0–4		
Neurological Exam				0.99	0.99–0.99
Mean ± SD	0.16 ± 0.5	0.15 ± 0.5	0.15 ± 0.5		
Range	0–4	0–4	0–4		
Total score					
Mean ± SD	0.63 ± 1.4	0.63 ± 1.4	0.63 ± 1.4	0.99	0.99–1.00
Range	0–8	0–8	0–8		

### MPSM score validity

**Figure 4:**
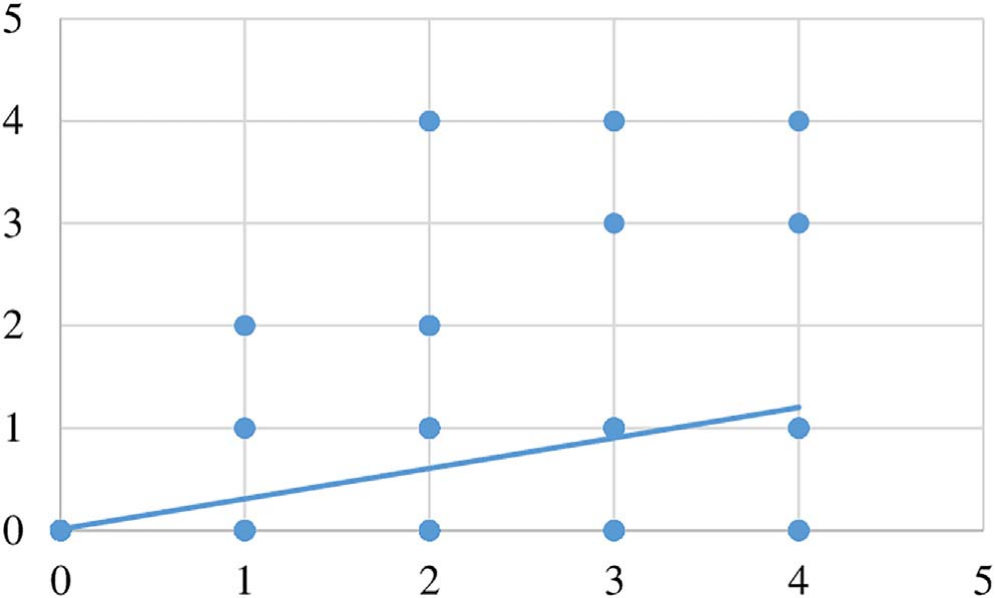
Correlation analysis observer I (*r* = 0.566, *P* = 0.000).

**Figure 5:**
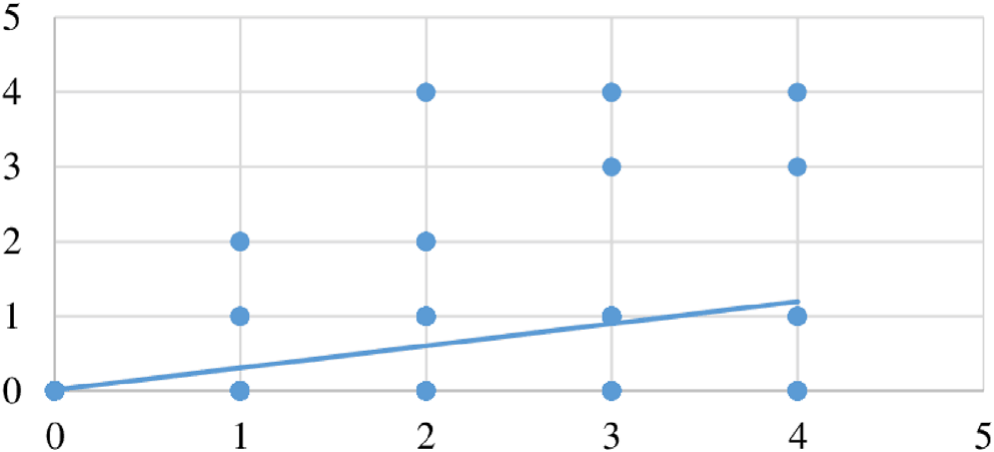
Correlation analysis observer II (*r* = 0.564, *P* = 0.000).

**Figure 6:**
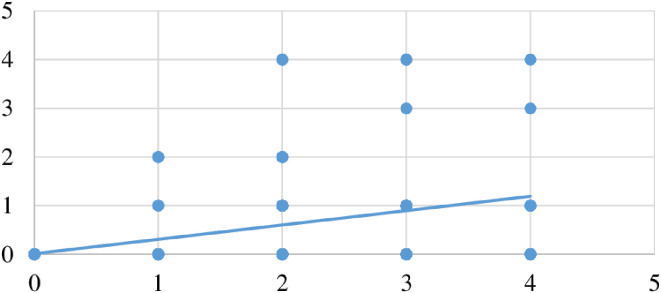
Correlation analysis observer III (*r* = 0.565, *P* = 0.000).

The convergent construct validity of the MPSM scoring system was examined by running a Pearson correlation analysis between the violation scores and neurological examination scores. A strong positive correlation was found where more severe neurological deficits were associated with greater degrees of screw-pedicle violation. The correlation was found to be statistically significant (Pearson test, *P* < .05) (Figures 4–6).

### Example of a score

**Figure 7:**
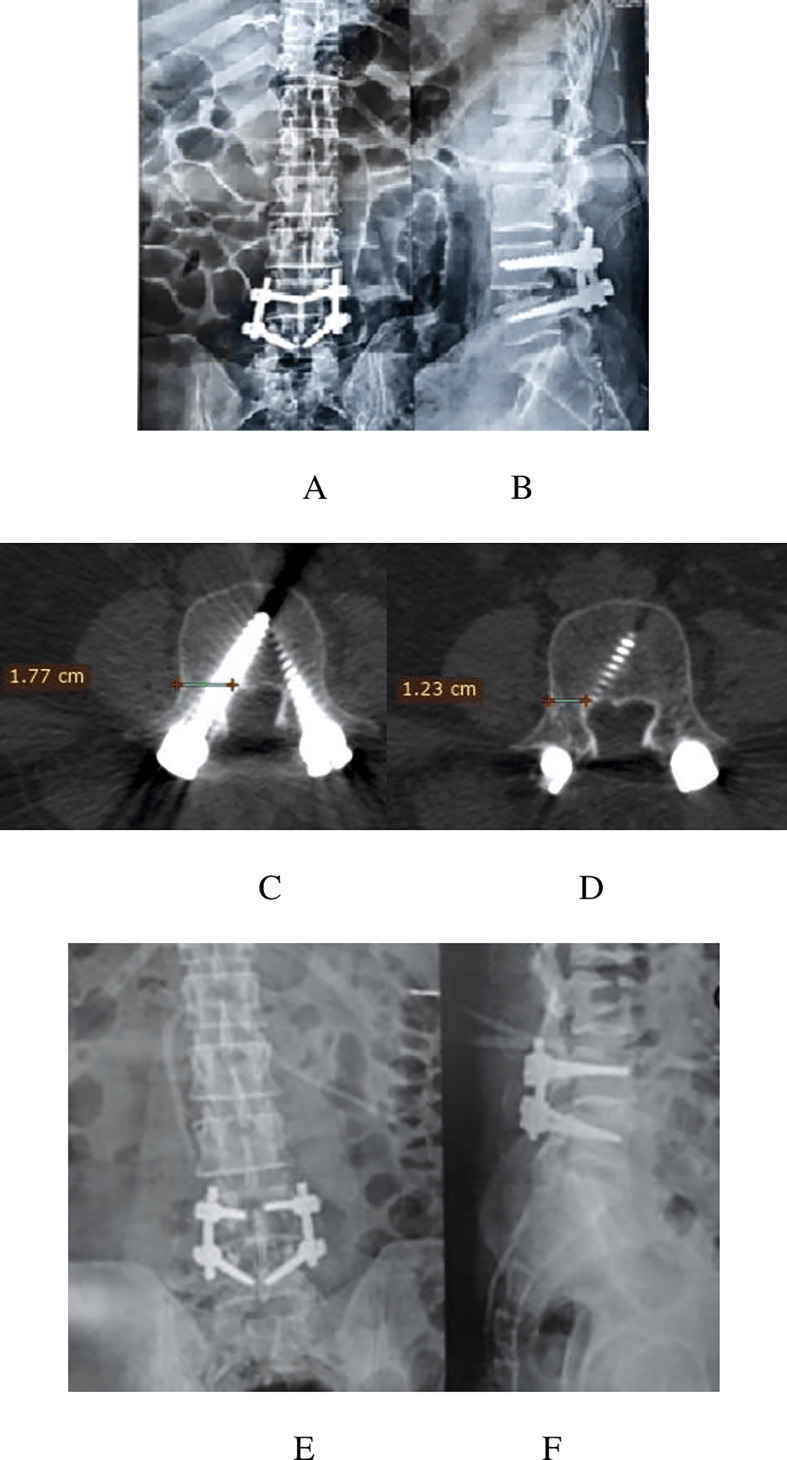
Plain X-ray images of the LSS AP (A), lateral (B), and axial (C) MSCT images showing a 5.4 mm medial violation of the L4 screw (measured by subtraction of 1.23 cm in Figure D from 1.77 cm in Figure C), plain X-ray images of the LSS AP (E) and lateral (F) after revision, and examination of the intraoperative medial wall of the pedicle and ensuring that no wall placed the screw. Therefore, there is no need for postoperative MSCT.

A 41-year-old male patient with LDP L4-5 underwent PLIF L4-5, a medial pedicular wall violation Rt L4, and developed Rt foot drop and severe pain according to the Rt L4 dermatome MPSM score 6. A revision was performed for RT L4, and after the revision, pain improved, but foot drop did not improve (Figure 7, Table 13).

**Table 13:** Data of the previous case according to the MPSM score and management decisions.

	Radiological grade	Neurological grade	MPSM score	Decision
Lt L4	0	0	0	No revision
Rt L4	3	3	6	Needs revision
Lt L5	0	0	0	No revision
Rt L5	0	0	0	No revision

## Discussion

In spine surgery, the precise placement of pedicle screws is essential for the effectiveness and stability of the surgical process as well as for reducing the possibility of iatrogenic damage to the anatomical structures surrounding the pedicle dural sac (medially), the superior and inferior nerve roots, and the vascular structures (anterolaterally). Anatomical landmarks, preoperative imaging, intraoperative imaging tools such as plain radiography, fluoroscopy, and, more recently, image-guided technologies can all be used as guides for instruments to ensure precision [11–14].

Attention to screw placement procedures has been focused on safety concerns, possible consequences from misplaced screws, and violations of pedicle walls. A significant amount of information has been gathered about the most effective way to interpret pedicle screw cortical breaches. The cortical breach has been characterized by using a variety of measures. When used in research from various institutions, these metrics have produced differing results. To ensure that the screw is inserted in the correct location and to locate the pedicle, fluoroscopic guidance is employed. However, postoperative CT scans are frequently needed, which are now recognized as the gold standard imaging technique for assessing the accuracy of pedicle screw placement Compared to plain X-ray which had lower sensitivity and specificity [9, 15–17].

In the present study, regarding screw misplacement, no statistically significant difference in terms of the direction or extent of screw violation was found between observers. There was a small space between the dural sac and pedicles at all levels, increasing from 1.29 mm at the L1 level to 1.56 mm at the L5 level. Medially placed screws may cause more nerve root irritation because they are separated from the dural sac by a thin layer of epidural fat, which is normally 2 mm thick.

In a study conducted by Castro et al., 131 screws were inserted under fluoroscopic supervision into 30 patients. Forty percent of the patients with (CT) images exhibited cortical penetration, and 29% indicated medial wall penetration [18]. Similarly, Laine et al. [19] reported that the screw malposition rate ranged from 28.1% to 39.9%. Furthermore, Lotfinia et al. [20] and Castro et al. [18] reported that 35.22% and 40%, respectively, of their patients had improper screw placement.

Furthermore, Aigner et al. [21] reported that 245 patients had 1688 pedicle screws implanted. Ten thousand seventeen screws, or 95.2% of the total, were categorized as Ia in Zdichavsky et al.’s classification [11], which revealed that 51 screws (4.8%) had misplacement; 19 screws were categorized as Ib, 2 as IIa, 14 as IIb, 3 as IIIa, and 13 as IIIb. Misplacement of screws occurred in 42 patients (17.1%) overall. According to the classification of Rao et al. [22], the pedicle wall was punctured by 75 screws or 7.0%. There were 46 small (4 mm) perforations in the screws. The lateral and medial cortices of the pedicles were both pierced by 48 and 27 screws, respectively.

After this classification, incorrect screw placements were found in 64 patients (26.1%). In 56 screws (5.2%), an anterior spinal cortical hole was discovered. Six screws had an anterior perforation greater than 2 mm, whereas 50 screws had anterior cortical perforations smaller than 2 mm. Six screws with anterior perforations greater than 2 mm were found in 1 patient each in the 5th and 7th thoracic vertebrae and 2 patients each in the 3rd lumbar and 1st sacral vertebrae.

Pedicle screw misplacement was assessed using other approaches. An approved technique for safe pedicle screw implantation with proven efficacy is fluoroscopically assisted pedicle screw placement, which also increases screw placement accuracy, particularly in the upper and middle thoracic spine. Compared to traditional methods, clinical and experimental studies have demonstrated that the use of image-guided navigation techniques dramatically decreases the rate of pedicle perforation and screw malpositioning [23].

The degree of extracortical screw violation was used by Gertzbein and Robbins [10] to describe cortical breaches on a scale known as the Gertzbein scale. Under this approach, screws that are completely contained within a pedicle and show no signs of a cortical breach are graded as Grade 0, while higher grades are assigned in breach lengths that are multiples of 2 mm, measured from the pedicle’s medial border. In its initial implementation, the scale was designed to evaluate simply the extent of spinal canal encroachment; lateral screws were not included in the graded categorization.

Nevertheless, new research has extended the original Gertzbein scale (modified Gertzbein) by using it in all directions where a cortical breach may occur. The application of this graded classification in the six probable directions of the cortical breach – anterior, lateral, medial, inferomedial, inferolateral, and superior – was first demonstrated in a more recent investigation. As a result, six distinct grades, ranging from 0 to 3, were assigned to each screw [10, 24, 25].

Youkilis et al. [4] conducted a follow-up study that slightly modified the previous classification to establish three distinct grades based on breach distance: grade 1 corresponds to screws that did not exhibit any evidence of a pedicle breach, grade 2 corresponds to screws that breached 2 mm or less, and grade 3 corresponds to screws that breached more than 2 mm.

With regard to the MPSM score, safe screw violation (grade I/no revision required) was reported in 439 (86.4%), 440 (86.6%), and 439 (86.4%) patients according to Observer I, II, and III, respectively. Dangerous screw violation (Grade II, revision may be required) was reported in 62 (12.2%), 61 (12%), and 62 (12.2%) patients according to Observer I, II, and III, respectively. Extremely dangerous screw violation (Grade III, must be revised) was reported in 7 (1.4%) patients, according to all three observers. No statistically significant difference was found between observers in terms of MPSM grade (χ^2^ test, *P* = 0.931). The MPSM demonstrated excellent (100%) intra-observer reliability for each observer regarding the violation score and total MPSM score. Additionally, the MPSM has demonstrated excellent (99%) inter-observer reliability regarding the violation score and total MPSM score. Regarding the validity of the MPSM score, the convergent construct validity of the MPSM was examined by performing a Pearson correlation analysis between the violation scores and neurological examination scores. We found a strong positive and statistically significant difference (Pearson test, *P* < 0.05) between severe neurological deficits and a high degree of screw-pedicle violation.

Our study’s limitations include the absence of a control group and the fact that it was a single-center, nonrandomized study because blinding was not feasible at our institution. Verification of these findings requires multicenter research and a bigger sample size. However, its strengths include being the first to use a comprehensive clinical-radiographic scoring system with precise recommendations for treating harmful pedicular screw violations.

## Conclusion

MPSM scoring is a valid and reliable system for evaluating pedicular screw violations and their possible neurological consequences in the thoracic and lumbosacral spine from D7 to S1. Moreover, the three grades obtained from the MPSM score help make clear decisions after management.

## Data Availability

The datasets used and analysed during the current study are available from the corresponding author upon request.
